# Comparative analysis of HKT genes in *Ipomoea pes-caprae* unveils conserved Na^+^/K^+^ symporter functions within the gene family

**DOI:** 10.3389/fpls.2025.1538669

**Published:** 2025-04-01

**Authors:** Zhonghua Guo, Jin Sun, Xingguang Chen, Hui Li, Sisi Liang, Fengying Liu, Tong Qu, Huaer Wang, Xueli Li, Zitong Ou, Haoran Feng, Jinbiao Ma, Sheng Wang, Lulu Wang, Boping Tang, Gang Wang, Yuan Qin, Yan Cheng

**Affiliations:** ^1^ Fujian Provincial Key Laboratory of Haixia Applied Plant Systems Biology, State Key Laboratory of Ecological Pest Control for Fujian and Taiwan Crops, College of Life Sciences, College of Plant Protection, Fujian Agriculture and Forestry University, Fuzhou, China; ^2^ Center for Genomics, School of Future Technology, Haixai Institute of Science and Technology, Fujian Agriculture and Forestry University, Fuzhou, China; ^3^ Key Laboratory of Biogeography and Bioresources in Arid Land, Xinjiang Institute of Ecology and Geography, Urumqi, China; ^4^ Department of Biochemistry, Microbiology and Immunology, University of Saskatchewan, Saskatoon, SK, Canada

**Keywords:** *Ipomoea pes-caprae*, halophytes, HKT, sodium-potassium transport, salt stress

## Abstract

The HKT protein family plays a vital role in plant responses to salt stress by mediating sodium (Na^+^) and potassium (K^+^) transport and maintaining Na^+^-K^+^ balance. *Ipomoea pes-caprae* (*IPC*), a pantropical creeping plant distributed along coastal regions in tropical and subtropical zones, exhibits exceptional salt tolerance. Understanding its salt tolerance mechanisms provides valuable insights for developing salt-tolerant crops and identifying candidate genes for genetic engineering. In this study, we identified two HKT genes, *IpcHKT1;1* and *IpcHKT1;2*, in *IPC*. Phylogenetic analysis with HKT genes from other *Ipomoea* species revealed that all analyzed species contain two HKT genes located adjacently on the same chromosome. Comparative analysis of conserved motifs and intron-exon structures indicated that, despite their close evolutionary relationship, the HKT genes in *IPC* may exhibit functional divergence. Promoter analysis showed that their regulatory regions are enriched with cis-elements associated with responses to biotic and abiotic stresses, hormonal signaling, and growth, highlighting functional diversity within the HKT family. Subcellular localization experiments demonstrated that *IpcHKT1;1* and *IpcHKT1;2* are ion transporters localized to the plasma membrane. Heterologous expression in yeast confirmed their role in Na^+^/K^+^ symporter. Furthermore, RT-qPCR analysis revealed distinct expression patterns under salt stress: *IpcHKT1;2* was significantly upregulated in roots, while *IpcHKT1;1* expression was transitionally downregulated at 400 mM NaCl treatment. Prolonged high expression of *IpcHKT1;2* in roots suggests its critical role in sustained salt stress tolerance. These findings provide new insights into the molecular mechanisms of salt tolerance in *IPC*. The identification of *IpcHKT1;1* and *IpcHKT1;2* as key players in salt stress responses offers promising genetic resources for enhancing crop resilience to soil salinity, addressing challenges associated with global salinization.

## 1 Introduction

Soil salinization, driven by climate change and human activities, is one of the most severe environmental challenges worldwide. It leads to a reduction in arable land and poses a significant threat to both the yield and quality of crops (Cheng et al., 2023; Schroeder et al., 2013). High concentrations of salts in agricultural soils result in the accumulation of reactive oxygen species (ROS) in plant cells, impairing the plant’s ability to absorb water and essential mineral nutrients, which in turn inhibits growth (Petrov et al., 2015). Consequently, the development of salt-tolerant crop varieties has been a major goal in global crop improvement efforts (Ashraf and Munns, 2022; Katerji et al., 2003; Shams and Khadivi, 2023; Shams et al., 2023). Potassium (K^+^) is a key cation in plant cells, comprising 2-10% of the dry weight of plants. It is essential for plant growth and development (Leigh and Wyn Jones, 1984), and its presence enhances the plant’s ability to adapt to a variety of biotic and abiotic stresses, including drought and salinity (Zörb et al., 2014). Sodium (Na^+^) is one of the most common soluble cations in saline-alkaline soils, and when its concentration exceeds a certain threshold, it induces ionic toxicity. Furthermore, Na^+^ disrupts the balance of K^+^ within the plant, causing cellular damage. Therefore, maintaining a high cytoplasmic K^+^/Na^+^ ratio is crucial for maintaining ion homeostasis within plant cells.

Plants tolerate sodium (Na^+^) toxicity through two main mechanisms: sodium exclusion and tissue tolerance (Zhu, 2003). Ion-selective transport capacity is a key factor determining a plant’s ability to adapt to saline environments, with K^+^/Na^+^ transporters playing a critical role in this process. Key transporters, such as the Na^+^/H^+^ antiporter (SOS1), Na^+^/H^+^ exchangers (NHXs), and high-affinity K^+^ transporters (HKTs), form a complex network that regulates the uptake, transport, and compartmentalization of Na^+^ (Van Zelm et al., 2020; Volkov, 2015). Thus, identifying and characterizing Na^+^/K^+^ transporters is crucial for understanding how plants maintain ion homeostasis and confer salt tolerance. Among the K^+^/Na^+^ transporters, the high-affinity K^+^ transporter (HKT) family plays a critical role in both plant mineral nutrition and salt stress regulation. The first HKT protein was cloned from wheat in 1994 (TaHKT2;1) (Schachtman and Schroeder, 1994a). Initially characterized as a high-affinity K^+^ transporter, HKT proteins were subsequently shown to transport other ions, such as Na^+^ (Rubio et al., 1995). Studies have demonstrated that HKTs are involved in regulating plant salt tolerance, and under conditions of severe K^+^ deficiency, they can facilitate Na+ uptake, providing an adaptive mechanism for coping with short-term K^+^ shortages.

The HKT (High-Affinity K^+^ Transporter) family belongs to the Trk/Ktr/HKT superfamily and is characterized by a distinct structure composed of four transmembrane domains, a pore domain, and additional transmembrane units (MPM1-MPM4) (Riedelsberger et al., 2021b). Phylogenetic analysis has divided the HKT family into two subgroups: Class I and Class II (Gomez-Porras et al., 2012; Haro et al., 2010; Platten et al., 2006). The primary difference between these subgroups lies in the presence of a serine residue (SGGG) or a glycine residue (GGGG) in the first pore loop (Mäser et al., 2002). Generally, Class I HKTs (SGGG) function as Na^+^ uniporters, while Class II HKTs (GGGG) mediate Na+ and K^+^ symport. However, within these two classes, there exists considerable functional diversity, which has yet to be fully explained at the molecular level. To date, multiple HKT genes have been identified and shown to play a role in salt tolerance across various species. In *Arabidopsis*, a single gene, AtHKT1, has been characterized, and its overexpression enhances salt tolerance by facilitating the transport of Na^+^ from the roots to the shoots (Berthomieu et al., 2003b; Mäser et al., 2002b; Rus et al., 2004). In rice, *OsHKT1;4* restricts the movement of Na^+^ from the leaf sheath to the leaves under salt stress, thereby improving salt tolerance. In maize, *ZmHKT1* transports Na^+^ in the xylem to promote salt tolerance, while *ZmHKT2* contributes to salt tolerance by regulating K^+^ levels in the (Cao et al., 2019; Jiang et al., 2018; Zhang et al., 2023). Mutation of the wheat TaHKT1 gene results in increased Na^+^ content in the leaves of transgenic lines, highlighting its role in regulating Na^+^ transport from the roots to the leaves (Byrt et al., 2014b). Collectively, these findings demonstrate the involvement of HKT genes in plant responses to salt stress.

The expression patterns of *HKT* genes in response to varying K^+^ and Na^+^ conditions further elucidate their functional roles. For example, in rice, Class I HKT gene, *OsHKT1;1* showed higher expression in the shoots compared to the roots, with increased expression in the roots under high Na+ conditions and decreased expression in the shoots. Additionally, *OsHKT1;1* expression increases in both the leaves and roots under high Na^+^ conditions. In barley, *HvHKT1;5* expression is elevated in the roots under low K^+^, high K^+^, or high Na^+^ conditions (Huang et al., 2020). In contrast, Class II HKT genes, such as *OsHKT2;1* and *OsHKT2;2* in rice, exhibit increased expression under low K^+^ or low Na^+^ conditions, but decreased expression under high K^+^ or high Na^+^ conditions (Oomen et al., 2012). In barley, *HvHKT2* expression increases in the leaf sheath, leaves, and roots under low K^+^ conditions, but decreases in the leaf sheath and roots under high Na^+^ conditions, while it increases in the leaves (Mian et al., 2011). These findings suggest that the HKT gene family plays a crucial role in enhancing plant salt tolerance and ion transport. However, the underlying molecular mechanisms contributing to the observed functional diversity remain unclear.

Halophytes, defined as plants capable of completing their life cycle in saline environments exceeding 200 mM NaCl, represent invaluable genetic reservoirs for deciphering plant salt adaptation mechanisms. Their unique ion homeostasis strategies, particularly through specialized transporters, provide critical insights for crop salt tolerance improvement (Cheeseman, 2015; Munir et al., 2022; Ungar, 2023). A paradigmatic example is *Suaeda salsa*, which employs a sophisticated Na^+^ compartmentalization strategy. In this species, SsHKT1;1 functions as a dual-affinity potassium transporter (Shao et al., 2014), Coordinating with SsSOS1 (plasma membrane Na^+^/H^+^ antiporter) and SsNHX1 (vacuolar Na^+^/H^+^ exchanger) to establish tissue-specific Na^+^ gradients (Wang et al., 2020). Similar mechanisms have been characterized in *Thellungiella salsuginea*, where TsHKT1;2 modulates root-shoot Na^+^ partitioning through xylem loading regulation (Ali et al., 2013, Ali et al., 2012). Despite these advances, the functional diversification of HKT transporters in coastal pioneer species remains underexplored. *Ipomoea pes-caprae* is a perennial vine belonging to the *Convolvulaceae* family and the *Ipomoea* genus. It is widely distributed along tropical and subtropical coastal regions and is highly regarded for its medicinal and ecological properties. Its exceptional tolerance to abiotic stresses has also attracted considerable scientific attention (Cheng et al., 2021; Liu et al., 2020; Zhang et al., 2018). Therefore, the identification of functional genes involved in the response of *IPC* to extreme environmental conditions, and the application of these gene resources to the genetic improvement of salt-tolerant crops, holds significant potential for agricultural advancements. A high-quality reference genome for *IPC* was provided by our previous genome sequencing efforts (Cheng, 2023), establishing a foundation for the study of its salt tolerance mechanisms. In the present study, bioinformatics approaches were employed to identify two members of the *HKT* gene family in the *IPC* genome. The chromosomal locations, gene duplication events, evolutionary relationships, gene structures, and cis-regulatory elements of these HKT genes were also investigated in *IPC* and several closely related *Ipomoea* species. Furthermore, real-time quantitative PCR (qPCR) was performed to analyze the expression profiles of *IpcHKT1;1* and *IpcHKT1;2* in different tissues, providing insights into their roles in salt stress responses. Additionally, a yeast heterologous expression system was utilized to validate the ability of *IpcHKT1;1* and *IpcHKT1;2* to co-transport Na^+^ and K^+^ ions. Collectively, these findings provide valuable information for further understanding the involvement of *IpcHKT1;1* and *IpcHKT1;2* in salt stress tolerance and offer insights into the functional characterization of these genes.

## 2 Materials and methods

### 2.1 Plant materials and growth conditions

*Ipomoea pes-caprae* seeds were collected from a beach in Changle, Fuzhou, Fujian Province, China (Latitude 25°54’33” N, Longitude 119°40’42” E) (Wang et al., 2023; Ye et al., 2023). Seeds were manually scarified and then soaked in distilled water at 37°C for germination. After 24 hours incubation, the germinated seeds were transferred to soil to grow 7 days, then the seedlings were carefully excavated, rinsed with distilled water, and then transfer to hydroponic culture in half-strength (1/2 MS) nutrient solution. *Nicotiana benthamiana* seeds used in this study were sourced from laboratory stocks. Seeds were sown in a 1:1 mixture of vermiculite and nutrient soil and grown in the same greenhouse. The plants were grown in a controlled greenhouse environment with a photoperiod of 16 hours light and 8 hours dark, relative humidity maintained between 30% and 50%, and a temperature of 28°C.

### 2.2 Identification and phylogenic analysis of HKT genes in *Ipomoea* species

The genome assembly and annotation for *Ipomoea pes-caprae* were obtained from the National Genomics Data Center of China (https://www.cncb.ac.cn/) under BioProject ID PRJCA020559 (Wang et al., 2022). Genome data for additional *Ipomoea* species are provided in **Supplementary Table 1**. Amino acid sequences of HKT proteins from *Arabidopsis thaliana* and *Oryza sativa* were used as queries to identify HKT candidates in *Ipomoea* species via Blastp searches. Candidate HKT proteins were analyzed for conserved domains using the Pfam database (http://pfam.xfam.org/), and proteins lacking essential domains were excluded from further analysis. The amino acid number, molecular weight, and isoelectric point (pI) of HKTs proteins, was acquired from ExPasy (http://web.expasy.org/protparam) (Ye et al., 2022).

Multiple sequence alignments of HKT amino acid sequences from *IPC*, *A. thaliana*, and *O. sativa* were performed using MAFFT (https://mafft.cbrc.jp/alignment/software/) with default parameters. Phylogenetic analysis was conducted using IQ-TREE with the maximum likelihood (ML) method, applying the (Q.plant+G4) substitution model (http://www.iqtree.org/doc/Substitution-Models) and 1,000 bootstrap replicates. Subcellular localization predictions for *IPC* HKT proteins were carried out using CELLO (http://cello.life.nctu.edu.tw/).

### 2.3 Gene structure, conserved motifs, transmembrane domains, and protein model prediction of *IpcHKT1;1* and *IpcHKT1;2*

Gene structure information for *IpcHKT1;1* and *IpcHKT1;2* was obtained from our genome assembly (Cheng, 2023). Visualization of gene structure and conserved motifs was performed using TBtools software (https://github.com/CJ-Chen/TBtools). Protein structure models for *IpcHKT1;1* and *IpcHKT1;2* were generated with AlphaFold2 (https://alphafold.ebi.ac.uk/), followed by model fitting analysis.

### 2.4 Cis-element analysis of *IpcHKT1;1* and *IpcHKT1;2* gene promoters

The 2 kb sequences upstream of the transcription start site (TSS) for each *IpcHKT* gene were extracted as putative promoter regions using TBtools software (https://github.com/CJ-Chen/TBtools). These promoter sequences were analyzed in the PlantCARE database (http://bioinformatics.psb.ugent.be/webtools/plantcare/html/) to identify potential cis-regulatory elements.

### 2.5 Expression analysis of *IpcHKT1;1* and *IpcHKT1;2*

Three-week-old *Ipomoea pes-caprae* plants grown hydroponically were treated with 400 mM NaCl by transferring them to the same hydroponic medium supplemented with 400 mM NaCl. Root, stem, and leaf samples were collected prior to treatment (considered as 0 h) and at 4, 12, 24, and 72 hours post-treatment for RNA isolation and subsequent gene expression analysis. Total RNA was extracted using the Hipure Plant RNA Mini Kit (Magen, PMC11-01), and cDNA was synthesized using HiScript II Q RT SuperMix (Vazyme, 7E731J3). RT-qPCR reactions were prepared with 2x ChamQ Blue Universal SYBR qPCR Mix (Vazyme, 7E164014), and amplification was conducted on a Real-time quantitative PCR instrument (Bio-Rad Laboratories, Inc, CFX96). RNA samples from different tissues collected at different time points were used for reverse transcription. 1 µg of RNA was converted to cDNA in a 60 µL reaction volume. The reaction system was 20 µL (cDNA 1 µL, Forward primer 0.5 µL, Reverse primer 0.5 µL, SYBR qPCR Mix 10 µL, Water 8 µL).The IpcUBQ gene was used as a reference for the RT-qPCR analysis. The expression levels of target genes at 4, 12, 24, and 72 hours post-treatment were calculated relative to the 0-hour time point using the 2^^-ΔΔCt^ method. The primers used for RT-qPCR are listed in **Supplementary Table 1**.

### 2.6 Tobacco transformation and subcellular localization analysis

The coding sequences (CDS) of *IpcHKT1;1* and *IpcHKT1;2* were cloned into the PENTER (**Supplementary Figure S1**) and then transferred into PCAMBIA2300-35S-EGFP-35S-Neo vector (**Supplementary Figure S2**) by LR reaction. To create pCAMBIA2300-*IpcHKT1;1*-EGFP and pCAMBIA2300-*IpcHKT1;2*-EGFP constructs, which express GFP-tagged fusion proteins. These GFP-tagged constructs were co-transformed with a pBI121-mCherry-*fABD2* construct, which encodes *fABD2* fused to the red fluorescent protein (mCherry) as a plasma membrane marker, into 4-week-old *Nicotiana benthamiana* leaves, following the method described by (Chakrabarty et al., 2007). Fluorescence signals were observed 24 hours post-transformation using a Leica TCS SP8X confocal microscope (Lecia https://www.leica.com/). Primers used for constructing gene expression vectors are listed in **Supplementary Table 1**. The vectors used in this study are all preserved in our laboratory.

### 2.7 Functional complementation assay of *IpcHKT* in yeast

Full-length CDS of IpcHKT1;1 and IpcHKT1;2 were cloned into the PYES2-NBT vector (**Supplementary Figure S3**) via infusion cloning (Accurate Biology, 2.5×OK Clone Master Mix A6A1124), using EcoRI and BamHI to digest the vector backbone. The resulting constructs, along with the control (PYES2-NBT), were transformed into the yeast deficit strains CY162, AXT3K, using the LiAc/PEG transformation method as described by Ito et al (Ito et al., 1983). Positive transformants were selected on Ura-selective medium (Minimal SD Base (Coolaber, PM341426600). DO Supplement (Coolaber, PM332027325). Yeast growth phenotypes were assessed by spotting serial dilutions of the yeast cultures onto arginine phosphate (AP) medium (8 mM phosphoric acid, 10 mM L-Arginine, 2 mM MgSO_4_, 0.2 mM CaCl_2_, 2% glucose, plus vitamins and trace elements, and 1.5% (w/v) agar, pH 6.5) supplemented with different concentrations of Na^+^ or K^+^. Growth under these conditions was used to evaluate the ion transport capabilities of IpcHKT1;1 and IpcHKT1;2. Primers used for vector construction are listed in **Supplementary Table 1**. Yeast strain lacking the main sodium transporters AXT3K (*Δena1:: HIS:: ena4, Δnha1:: LEU2, Δnhx1:: KanMX4*) (Quintero et al., 2002), strain lacking potassium uptake proteins CY162 (*MATa, Δtrk1, trk2::pCK64, his3, leu2, ura3, trp1, ade2*) (Anderson et al., 1992), and control strain W303 (*W303-1B, MATα, ura3-1, leu2-3, 112 his3-11, 15 trp1-1, ade2-1, can1-100*) were kindly provided by Professor Ma Jingbiao at Xinjiang Institute of Ecology and Geography, Chinese Academy of Sciences (Haxim et al., 2023). PYES2-NBT vector was obtained from Fujian Academy of Agricultural Sciences (https://www.faas.cn/), and the detailed information about the vector was provided in **Supplementary Figure S3**.

## 3 Result

### 3.1 Physicochemical properties and phylogenetic analysis of the HKT family in *Ipomoea pes-caprae* and related species

**Figure 1:**
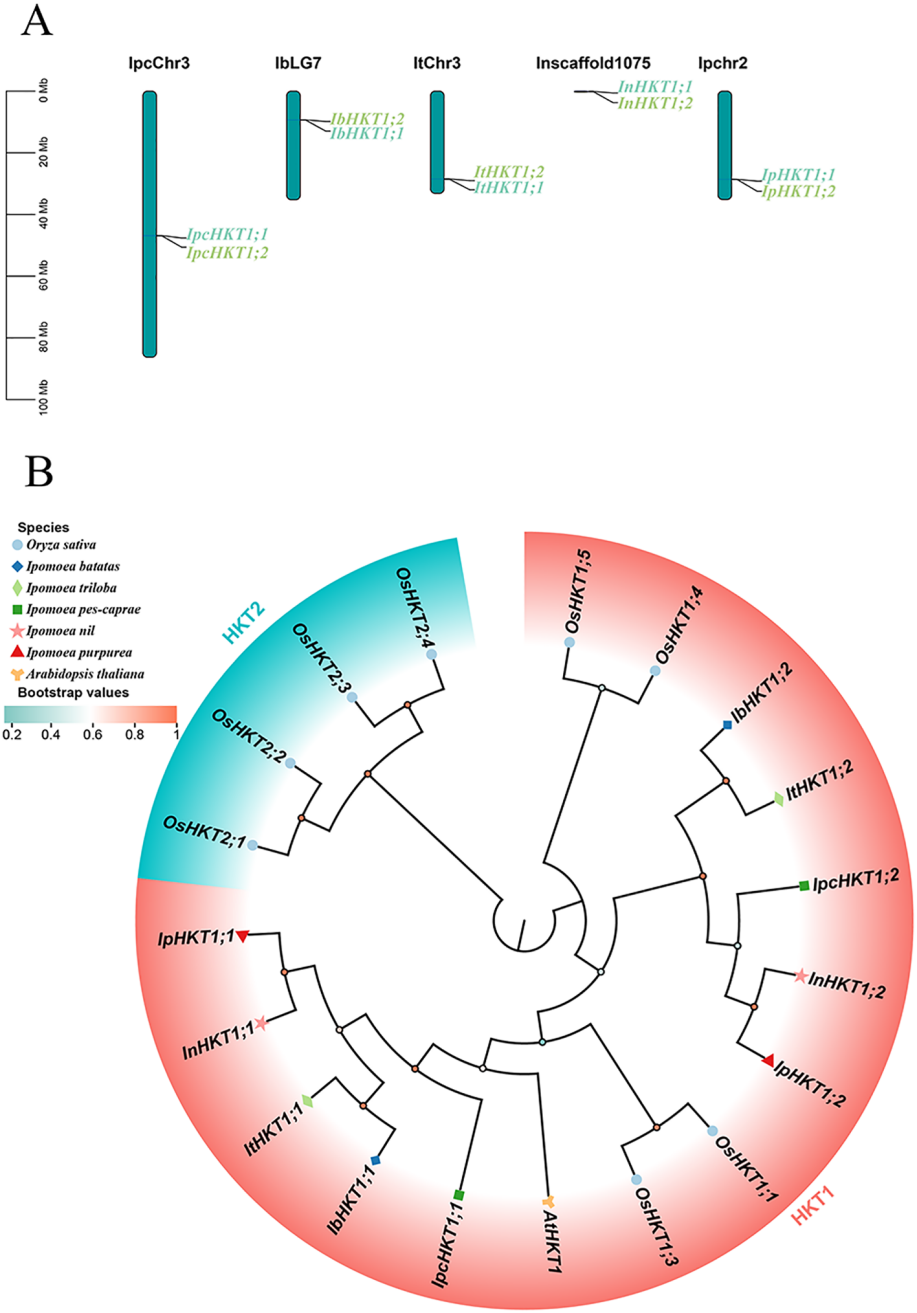
Chromosomal localization and phylogenetic analysis of HKT genes in the *Ipomoea* genus. HKT genes from five *Ipomoea* species, including *Ipomoea pes-caprae*, *Ipomoea batatas*, *Ipomoea triloba*, *Ipomoea nil*, and *Ipomoea purpurea*, were analyzed. **(A)** Chromosomal localization of HKT genes across the genomes of the respective species. **(B)** Phylogenetic relationships of *Ipomoea* HKT proteins, with HKT sequences from Arabidopsis and Rice included as outgroup references.

To understand the salt tolerance mechanisms in *Ipomoea pes-caprae* from the perspective of Na^+^ transport, we performed a genome-wide identification of HKT genes in this species. Two HKT genes were identified in the *IPC* genome. Interestingly, these two genes are located on the same chromosome, positioned in close proximity to each other (**Figure 1A**). In contrast, the model plant *Arabidopsis thaliana* has only one HKT gene, suggesting that the tandem arrangement of the two HKT genes in *IPC* may have resulted from a tandem duplication event during evolution. To test this hypothesis, we identified and analyzed the chromosomal locations of HKT genes in four additional sequenced species of the genus *Ipomoea*. A total of eight HKT family members were identified, with each *Ipomoea* species containing two HKT genes. Notably, the chromosomal locations of HKT genes in these species mirrored the pattern observed in *IPC*, with both genes situated on the same chromosome and closely linked (**Figure 1A**). This finding suggests that a tandem duplication event of HKT genes likely occurred during the evolutionary history of the *Ipomoea* genus, possibly originating from a common ancestor.

To further characterize these HKT genes, we analyzed the physicochemical properties of their encoded proteins. The molecular weights of *Ipomoea* HKT proteins varied from 39.9 kDa (IpHKT1;1) to 62.43 kDa (IpcHKT1;2), and their isoelectric points (pI) ranged from 8.54 (IpcHKT1;1) to 9.88 (ItHKT1;2) (**Table 1**). These results indicate a significant diversity in the physicochemical properties of HKT proteins within the *Ipomoea* genus. Subcellular localization predictions suggest that all identified *Ipomoea* HKT proteins are located in the plasma membrane, indicating a potential role in the plasma membrane that is consistent with the known function of HKT proteins in regulating ion transport across membranes.

**Table 1:** HKT genes and HKT proteins in *Ipomoea* species.

Gene	Species	pI	Mw	Chr	Start	End	Subcellular location
*IbHKT1;2*	*I. batatas*	9.51	47.3462	LG7	9308581	9313328	PlasmaMembrane
*IbHKT1;1*	*I. batatas*	9.75	44.3	LGT	9390163	9392424	PlasmaMembrane
*IpcHKT1;1*	*I. pes-caprae*	8.54	54.03	Chr3	46817441	46822663	PlasmaMembrane
*IpcHKT1;2*	*I. pes-caprae*	9.2	47.33	Chr3	46938813	46950231	PlasmaMembrane
*IpHKT1;1*	*I. purpurea*	9.01	39.9	chr2	28589753	28590835	PlasmaMembrane
*IpHKT1;2*	*I. purpurea*	9.72	60.55	chr2	28637257	28641277	PlasmaMembrane
*ImHKT1;1*	*I. nil*	9.79	56.41	scaffold1075	64150	71988	PlasmaMembrane
*InHKT1;2*	*I. nil*	9.74	62.43	scaffold1075	151924	156752	PlasmaMembrane
*ItHKT1;2*	*I. triloba*	9.88	62.18	Chr3	28448370	28453368	PlasmaMembrane
*ItHKT1;1*	*I. triloba*	9.75	57.24	Chr3	28576371	28582260	PlasmaMembrane

This **Supplementary Table** Summarizes the names and chromosomal locations of HKT genes, as well as the biochemical properties and subcellular localizations of the corresponding HKT proteins in *Ipomoea* species.

To gain further insight into the evolutionary relationships of *Ipomoea* HKT genes, a maximum likelihood (ML) phylogenetic tree was constructed, incorporating HKT genes from *Ipomoea* species, *Arabidopsis thaliana*, and rice (**Figure 1B**). Phylogenetic analysis showed that HKT family members are divided into two classes: Class I and Class II. However, all *Ipomoea* HKT genes were classified as Class I, suggesting a shared evolutionary origin and functional characteristics within the genus. Additionally, the two *Ipomoea* subgroups HKT1;1 and HKT1;2 clustered closely within the phylogenetic tree, indicating a close evolutionary relationship between them. This clustering further implies a high degree of relatedness among the HKT genes in the *Ipomoea* genus.

### 3.2 Structural analysis of IpcHKT1;1 and IpcHKT1;2 genes

**Figure 2:**
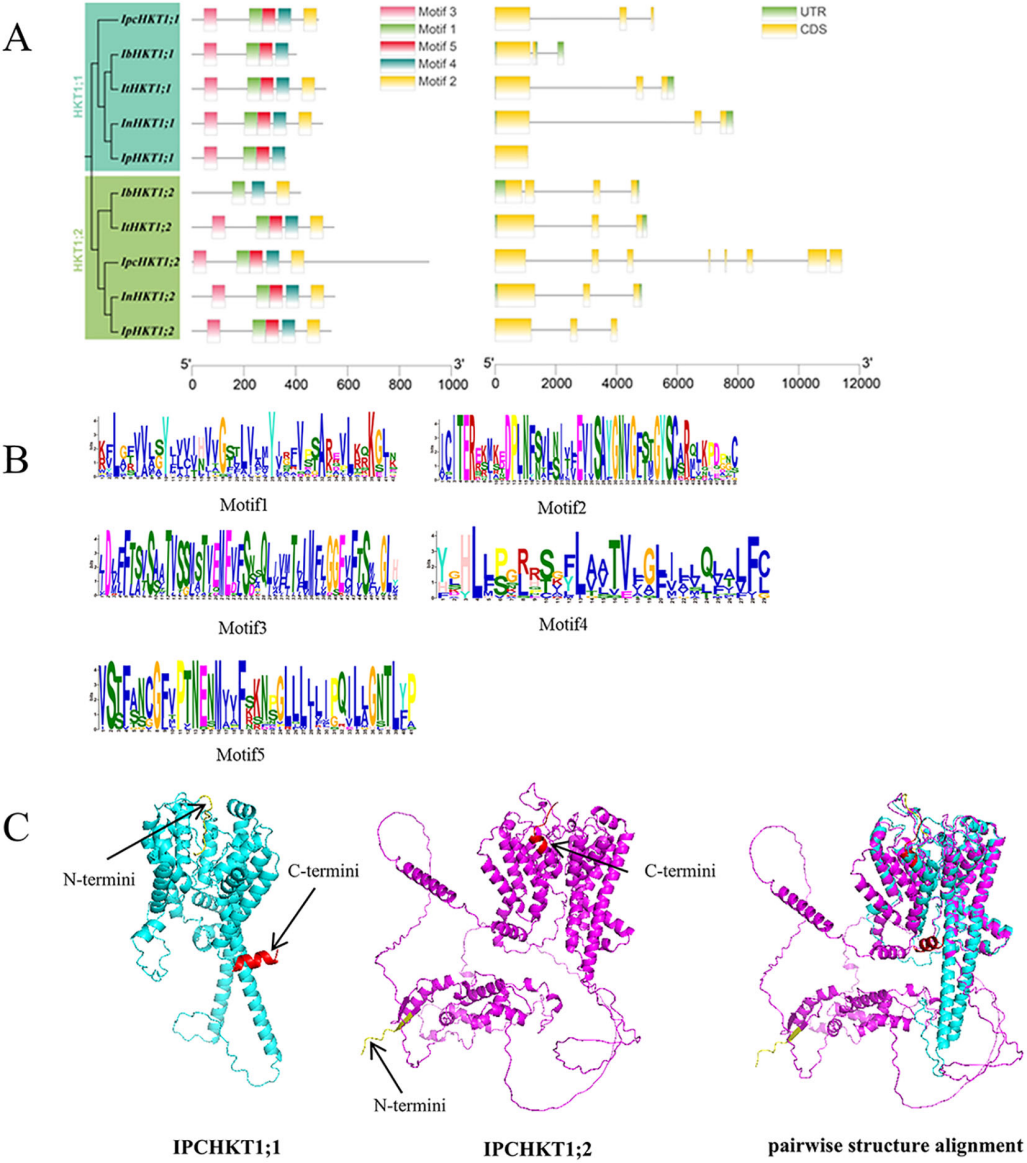
Gene structure and conserved domains of HKT genes in various *Ipomoea* species. **(A)** The conserved domains (left) of HKT proteins and the gene structure of HKT genes (right) are shown, with genes listed in phylogenetic order. **(B)** Conserved amino acids and their frequencies are displayed for each motif. **(C)** 3D structures and pairwise structure alignment of IpcHKT1;1 and IpcHKT1;2. The amino acids marked in red represent the C-terminus, and the amino acids marked in yellow represent the N-terminus.

To understand the gene splicing and protein characteristics of HKTs, we investigated the gene structure and conserved protein motifs of HKT family members in *Ipomoea* species. Gene structure analysis revealed that, except for *IpHKT1;1*, which has only one exon, the other *Ipomoea* HKT genes are composed of three exons, indicating a high degree of structural conservation in HKT genes within the *Ipomoea* genus. Conserved motif analysis showed that, with the exception of *IbHKT1;2*, *IbHKT1;1*, and *IpHKT1;1*, most *Ipomoea* HKT proteins contain five conserved motifs (motifs 1–5). The *IbHKT1;2* protein lacks motifs 3 and 5, while *IbHKT1;1* and *IpHKT1;1* lack motif 2 (**Figures 2A, B**). Notably, both *Ipomoea pes-caprae* HKT proteins contain all five motifs, indicating a relatively higher degree of conservation in *IPC* HKT proteins within the genus.

Further protein structure modeling using AlphaFold showed that *IpcHKT1;1* and *IpcHKT1;2* have a highly conserved core region, while the N- and C-terminal regions exhibit structural differences (**Figure 2C**). The high consistency in the core regions suggests functional conservation and possible redundancy of HKT proteins, while the structural differences in the N- and C-terminal regions imply potential functional diversification.

### 3.3 Cis-acting element analysis suggests HKT genes may participate in stress responses and plant growth and development

**Figure 3:**
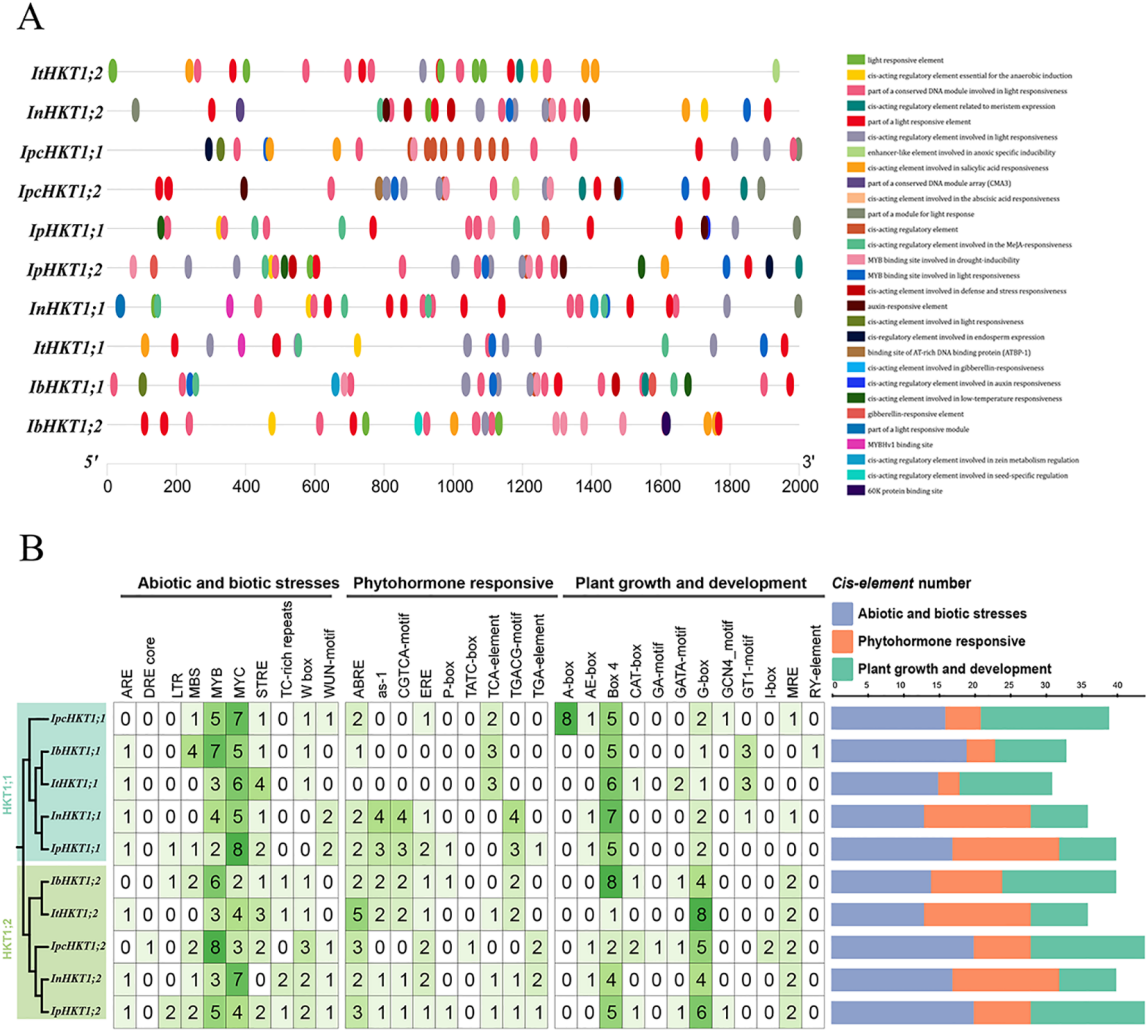
Cis-acting elements in the promoter regions of HKT genes from five *Ipomoea* species. **(A)** Distribution of cis-acting elements within the promoter regions of *HKT* genes, represented by colored rectangles. **(B)** Quantification and analysis of cis-acting elements in the promoter regions of *HKT* genes from *Ipomoea pes-caprae*, *Ipomoea batatas*, *Ipomoea triloba*, *Ipomoea nil*, and *Ipomoea purpurea*.

To explore the functional roles of HKT genes from a gene expression regulation perspective, we performed a cis-acting element analysis on the upstream sequences of *Ipomoea* HKT genes. As shown in **Figure 3A**, the promoter regions of *Ipomoea* HKT genes are rich in various cis-elements associated with biotic and abiotic stress responses, hormone response, and growth and development (**Figure 3B**). For biotic and abiotic stress responses, MYB and MYC elements were found to be common in the promoters of *Ipomoea* HKT genes, followed by STRE and MBS elements. Some genes, such as *InHKT1;2*, lack the STRE element, while *InHKT1;1* and *ItHKT1;1* are missing the MBS element. Notably, *IpcHKT1;2* in *IPC* contains the highest number of MYB elements (8), while *IpcHKT1;1* has more MYC elements (7). Previous studies have reported that MYB and MYC transcription factors are widely involved in salt-stress induction, suggesting that *IPC* HKT genes may be upregulated in response to salt stress and play roles in salt-stress adaptation. In terms of hormone response, ABRE elements are prevalent across *Ipomoea* HKT genes, with all genes, except *ItHKT1;1*, containing this element in their promoters. The promoters of *IpcHKT1;1* and *IpcHKT1;2* contain relatively fewer hormone-responsive cis-elements. Both promoters include ABRE and ERB elements; however, *IpcHKT1;1* also contains a TCA-element, while *IpcHKT1;2* has more TGA-elements and a unique TATC-box (one copy only). Regarding growth and development-related elements, Box4 and G-box elements are widely distributed in each gene’s promoter region, with Box4 elements being the most abundant. Interestingly, the promoter of *IpcHKT1;1* contains five Box4 elements and two G-box elements, while *IpcHKT1;2* shows the opposite distribution, with two Box4 elements and five G-box elements. Additionally, only the *IpcHKT1;1* promoter region contains eight A-box elements, which are absent in other genes. These findings suggest that *IpcHKT1;1* and *IpcHKT1;2* are broadly involved in plant response mechanisms, including biotic and abiotic stress responses, hormone signaling, and regulation of plant growth and development.

### 3.4 Subcellular localization analysis indicates that *IPC* HKT proteins function primarily in the plasma membrane

**Figure 4:**
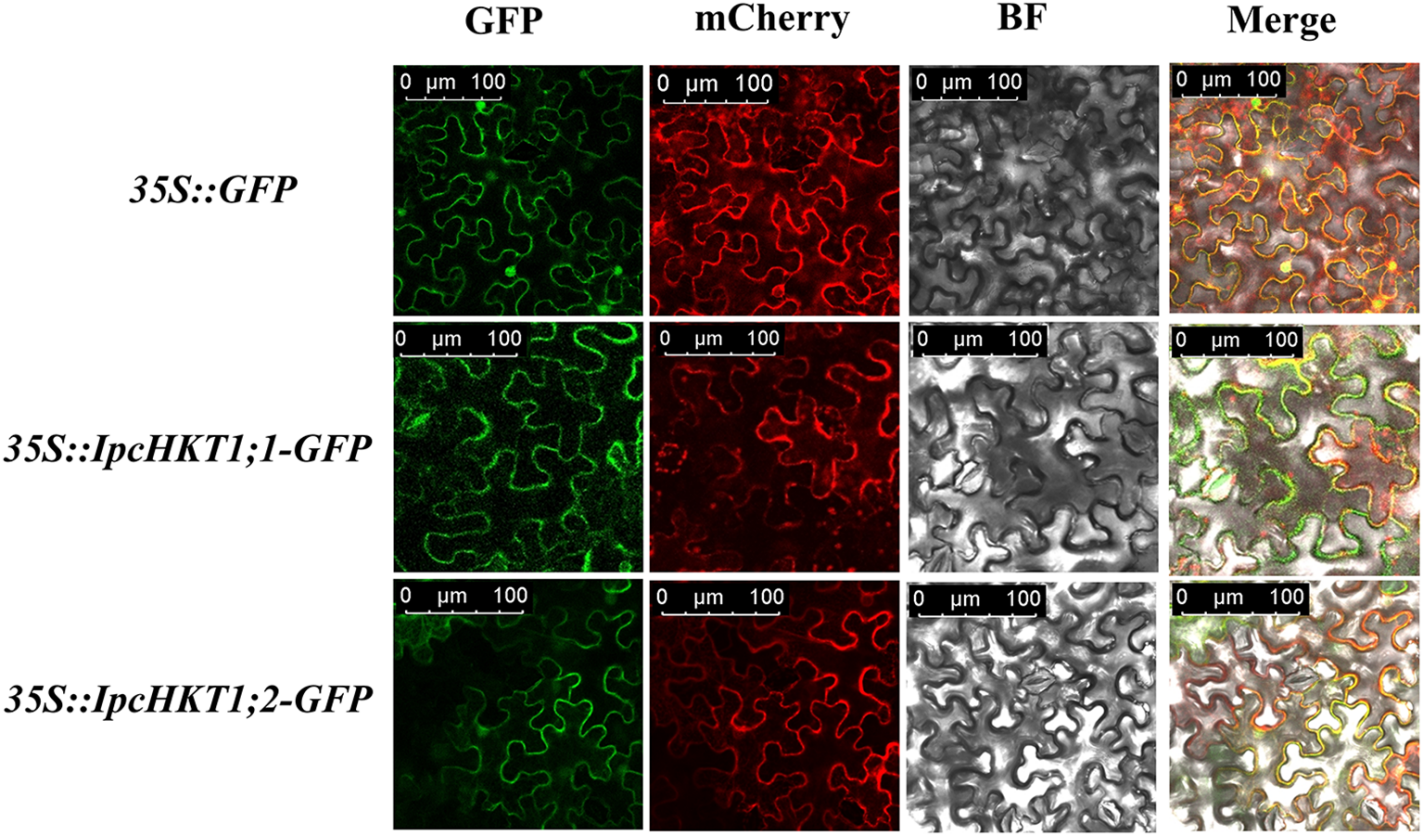
Subcellular localization of HKT proteins in *Ipomoea pes-caprae*. Localization of GFP-tagged *IpcHKT1;1* and *IpcHKT1;2* proteins, co-expressed transiently with an mCherry protein targeting the plasma membrane, in *Nicotiana benthamiana* leaf epidermal cells. Scale bars represent 100 µm in all panels.

Previous studies have shown that plant HKT proteins typically localize to the plasma membrane of plant cells, where they play a role in the transport of potassium (K^+^) and sodium (Na^+^). In this study, structural predictions for *IpcHKT1;1* and *IpcHKT1;2* indicated that both proteins are primarily localized in the plasma membrane. To confirm this prediction, we constructed green fluorescent protein (GFP) fusion expression constructs for *IpcHKT1;1* and *IpcHKT1;2*, which were transiently expressed in *Nicotiana benthamiana* cells. GFP signal visualization allowed us to observe the distribution of *IpcHKT1;1* and *IpcHKT1;2* in the epidermal cells of tobacco leaves. The results showed that the GFP signals for both *IpcHKT1;1-GFP* and *IpcHKT1;2-GFP* were localized at the cell periphery, consistent with plasma membrane localization (**Figure 4**). To further validate this result, we co-expressed a plasma membrane marker, fABD2-mCherry (red fluorescent protein), as a reference for plasma membrane positioning. Under confocal microscopy, the green fluorescence from *IpcHKT1;1-GFP* and *IpcHKT1;2-GFP* colocalized with the red fluorescence of fABD2-mCherry at the plasma membrane, confirming that *IpcHKT1;1* and *IpcHKT1;2* are indeed localized to the plasma membrane. This finding suggests that *IpcHKT1;1* and *IpcHKT1;2* function at the plasma membrane, consistent with their roles as ion transport proteins.

### 3.5 Expression analysis of *IpcHKT1;1* and *IpcHKT1;2* genes under salt stress

**Figure 5:**
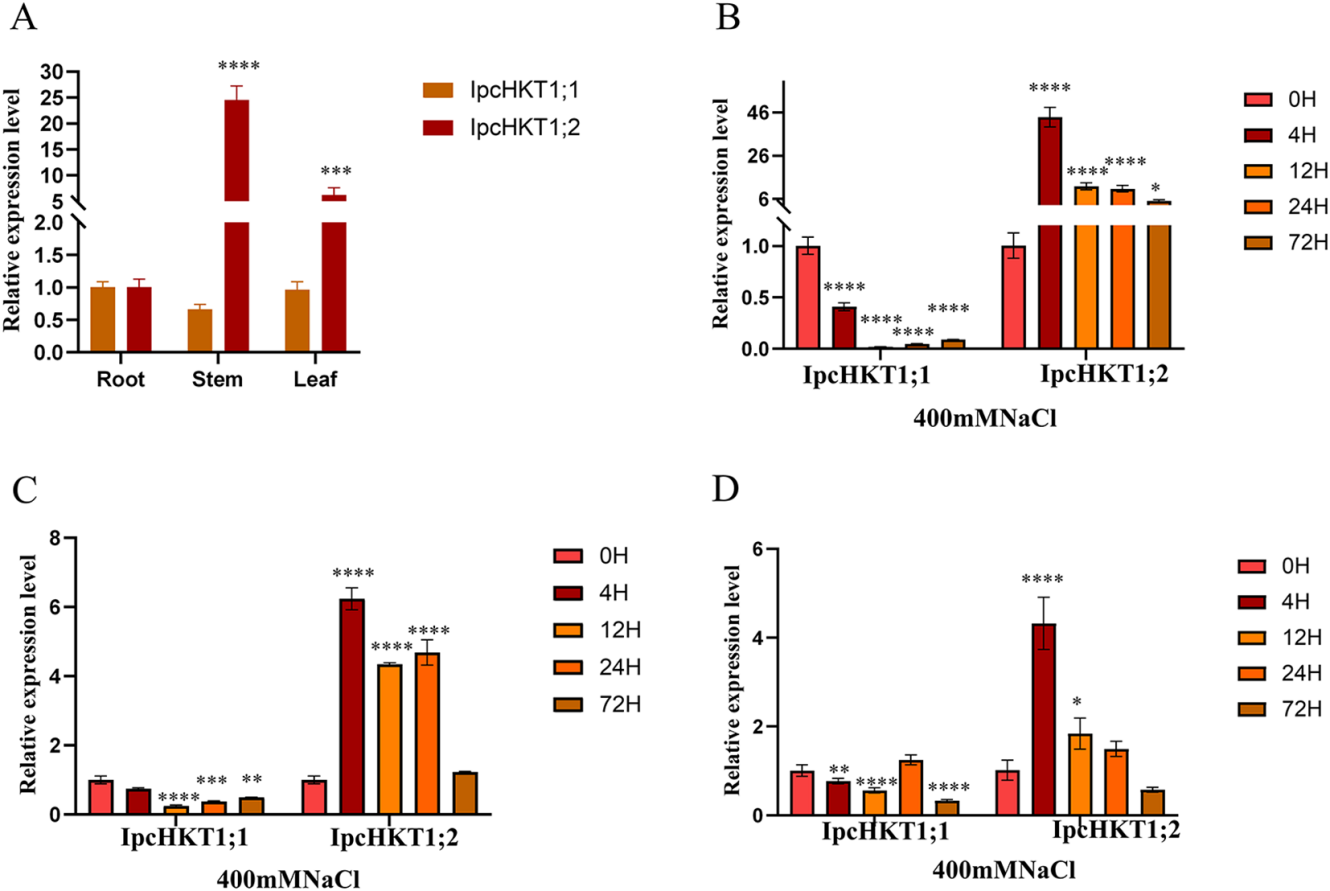
Expression profiling of HKT genes in *Ipomoea pes-caprae*. **(A)** Expression levels of *IpcHKT1;1* and *IpcHKT1;2* in roots, stems, and leaves under normal conditions are shown. Asterisks indicate significant differences in expression level between roots, leaves and stems. **(B–D)** Expression levels of *IpcHKT1;1* and *IpcHKT1;2* and in roots **(B)**, stems **(C)**, and leaves **(D)** following treatment with 400 mM NaCl at 0, 4, 12, 24, and 72 hours. The *IpcUBQ* gene was used as a reference for the RT-qPCR analysis. The expression levels of target genes at 4, 12, 24, and 72 hours post-treatment were calculated relative to the 0-hour time point using the 2^^-ΔΔCt^ method. The primers used for RT-qPCR are listed in **Supplementary Table 1**. Asterisks indicate significant differences between each time point and 0 h as indicated by t-test (*P<0.05, **P<0.01, ***P<0.001, ****P<0.0001).

We analyzed the expression profiles of *IPC* HKT genes. Under normal conditions, both *IpcHKT1;1* and *IpcHKT1;2* genes are expressed in various tissues. For *IpcHKT1;1*, expression levels are highest in roots, followed by leaves and stems, while *IpcHKT1;2* shows highest expression in stems, followed by leaves and roots (**Figure 5A**). Under 400 mM NaCl stress, RT-PCR analysis across tissues revealed that *IpcHKT1;1* expression remained relatively low in roots, stems, and leaves, showing a declining trend over time. In contrast, *IpcHKT1;2* exhibited higher expression levels across tissues, with a distinct stress response pattern: an initial upregulation followed by a gradual decline (**Figures 5B–D**). Notably, *IpcHKT1;2* expression in roots was significantly higher than in stems and leaves, demonstrating a pronounced response to salt stress in this tissue. Specifically, root expression of *IpcHKT1;2* was upregulated more than forty-fold within 4 hours of salt treatment, then decreased to about six-fold the baseline by 12 hours, stabilizing at this level over subsequent time points. In summary, both *IpcHKT1;1* and *IpcHKT1;2* are expressed across tissues in *IPC*; under salt stress, *IpcHKT1;1* shows an initial downregulation followed by gradual recovery, whereas *IpcHKT1;2* is upregulated across tissues, particularly in roots where it exhibits a rapid stress response. These results suggest that *IpcHKT1;2* may play a key role in *IPC*’s response to salt stress.

### 3.6 Functional evaluation of *IpcHKT1;1* and *IpcHKT1;2* in a yeast recombinant system

**Figure 6:**
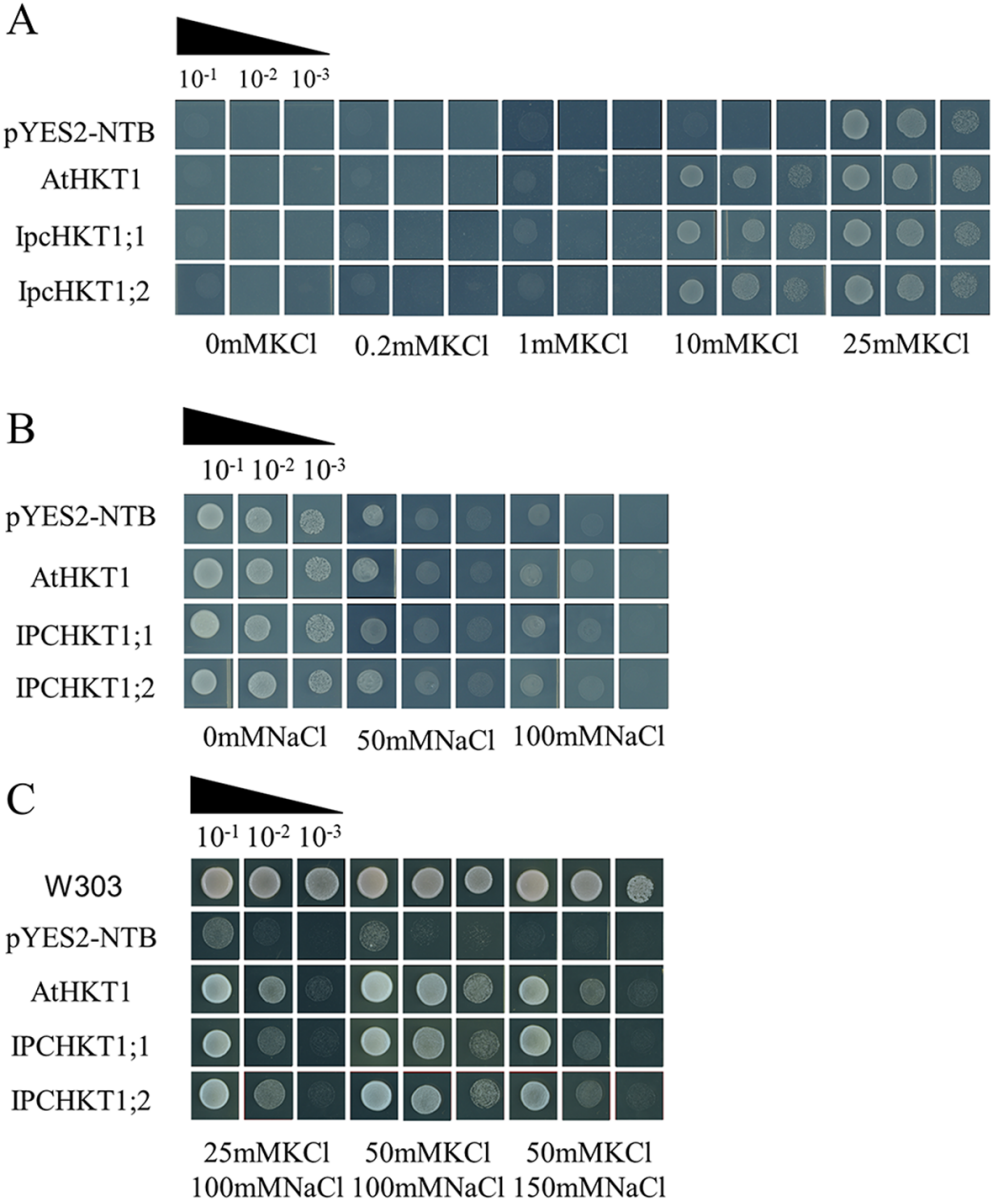
Functional complementation assay of *IpcHKT1;1* **and** *IpcHKT1;2* **in defective yeast strains.** The constructs expressing *IpcHKT1;1*, *IpcHKT1;2*, and *AtHKT1* using the pYES2-NBT vector backbone were introduced into CY162 (potassium uptake-deficient mutant yeast strain), AXT3K (salt-sensitive mutant yeast strain), and W303 (control strain) to assess their K^+^ and Na^+^ transport capacities. **(A)** Growth of CY162 yeast expressing the indicated HKT proteins on AP media supplemented with varying concentrations of KCl. **(B)** Growth of AXT3K yeast expressing the indicated HKT proteins on AP media supplemented with varying concentrations of KCl. **(C)** Growth of AXT3K yeast expressing the indicated HKT proteins on AP media supplemented with different combinations of KCl and NaCl. W303 was used as the wild-type control strain (first row).

To confirm the ion transport functions of *IPC* HKT proteins, we performed functional complementation assays in yeast mutant strains. First, we heterologously expressed *IpcHKT1;1* and *IpcHKT1;2* in the potassium uptake-deficient yeast mutant CY162 to examine their role in potassium ion transport. Similar to control yeast transformed with the empty vector (PYES2-NBT), yeast expressing *Arabidopsis thaliana AtHKT1*, *IpcHKT1;1*, or *IpcHKT1;2* failed to grow in media with less than 10 mM K^+^. However, at 10 mM K^+^, yeast expressing *AtHKT1*, *IpcHKT1;1*, or *IpcHKT1;2* could grow, while the control could not. At K^+^ concentrations exceeding 10 mM, all constructs supported yeast growth (**Figure 6A**). These findings indicate that *IpcHKT1;1* and *IpcHKT1;2*, like *AtHKT1*, do not function as strong K^+^ transporters but exhibit weak K^+^ transport activity. Next, we expressed the constructs (PYES2-NBT, *AtHKT1*, *IpcHKT1;1*, and *IpcHKT1;2*) in the salt-sensitive yeast mutant AXT3K to assess salt tolerance under different NaCl concentrations. At 50 mM NaCl, both control and experimental transformants grew well. However, at 50 and 100 mM NaCl, the yeast strains expressing *AtHKT1*, *IpcHKT1;1*, or *IpcHKT1;2* exhibited slightly stronger growth compared to the control strain, although the difference was not statistically significant (**Figure 6B**). Further, we supplemented the 100 mM NaCl medium with 25 mM and 50 mM KCl. In the AXT3K background, the control transformant failed to grow, while yeast expressing *AtHKT1*, *IpcHKT1;1*, or *IpcHKT1;2* showed improved growth as KCl concentrations increased (**Figure 6C**). This suggests that KCl addition significantly enhances the Na^+^ transport ability of *HKT1;2*, increasing NaCl tolerance in the AXT3K yeast mutant. With 50 mM KCl, AXT3K yeast expressing *AtHKT1*, *IpcHKT1;1*, or *IpcHKT1;2* tolerated up to 150 mM NaCl (**Figure 6C**). These results indicate that *IPC IpcHKT1;1* and *IpcHKT1;2* function similarly to *A. thaliana AtHKT1*, primarily as co-transporters of sodium and potassium ions.

## 4 Discussion

The increasing frequency of extreme environmental conditions such as high temperatures, freezing, drought, flooding, and high salinity has had a significant impact on global agricultural production and food security. Among these, soil salinization is a challenging environmental issue affecting 20% of the world’s irrigated land (Ali and Salem, 2024; Mukhopadhyay et al., 2021; Saifullah et al., 2018; Ventura et al., 2015). Excessive soil salinization not only reduces the productivity of salt-sensitive crops, such as sweet potatoes, wheat, and corn, but also decreases the productivity of salt-tolerant crops, such as cotton, barley, and sugar beet. By 2025, drought and salinization are projected to affect 50% of arable land (Wang et al., 2003). Studies have shown that the combination of high temperatures and high salinity has a detrimental effect on root growth in plants. In tomatoes, plants grown under high salinity, high temperature, or a combination of both showed reduced root growth (Rivero et al., 2014). A similar effect was observed in barley, where the combination of high temperature and salt stress inhibited root growth more than salt stress alone (Faralli et al., 2015). In wheat, seedlings subjected to both heat and salt stress showed greater inhibition of root growth compared to plants treated with salt alone (Keleş and Öncel, 2002) (Hamada and Khulaef, 1995). Quinoa also exhibited greater reductions in growth and yield under the combined stresses of drought, salinity, and high temperature compared to individual stressors (Abbas et al., 2023). These reports suggest that adverse environments, such as high salinity and high temperature, can cause severe damage to plant growth, particularly root development. However, *IPC*, a vine species that grows along beaches, is capable of developing highly developed roots even in high-temperature and high-salinity environments (Grigore and Vicente, 2023). Therefore, studying this plant is of significant importance for improving the ability of sweet potatoes and other crops to withstand high-temperature and high-salinity stresses.

The HKT family of proteins has been reported to regulate various developmental and stress responses in plants (Riedelsberger et al., 2021a). Previous studies have indicated that HKT family genes control the concentration and balance of K^+^ within plant cells. To date, members of the HKT family have been identified in various species, including *Arabidopsis thaliana* (Berthomieu et al., 2003a; Chandran et al., 2023; Mäser et al., 2002a; Rus et al., 2004), *Oryza sativa L* (Imran et al., 2021; Jabnoune et al., 2009; Oomen et al., 2012; Wang et al., 2024), *Zea mays L* (Cao et al., 2021; Jiang et al., 2018; Zhang et al, 2023), *Triticum aestivum L* (Byrt et al., 2014a), and Hordeum vulgare L (Huang et al., 2020; Mian et al., 2011). In this study, we identified 10 HKT family members across 5 species within the *Ipomoea* genus, including *IPC*. We found that the number of HKT genes in species of the *Ipomoea* genus was consistent, with only two members present, located on the same chromosome and tightly linked. This suggests that the HKT genes in the *Ipomoea* genus share a common evolutionary origin. It is likely that the ancestral species of *Ipomoea* underwent a tandem duplication event in its evolutionary history, and this characteristic has been retained across different *Ipomoea* species, indicating that this tandem duplication event may have conferred an adaptive advantage for regulating and enhancing the function of HKT genes in plants.

Further analyses of the physicochemical properties, gene structure, and cis-regulatory elements suggest that the functions of the HKT genes in the *Ipomoea* genus may differ. For example, the promoter region of *IpcHKT1;1* contains five Box4 elements and two G-box elements, while *IpcHKT1;2* has the opposite configuration, with two Box4 elements and five G-box elements. In addition, the promoter region of *IpcHKT1;1* uniquely contains eight A-box elements, which are absent in other HKT genes (**Figure 3B**). It has been reported that A-box elements in the *Solanum lycopersicum* DREB family cis-elements may play a role in responding to salt and heat stress (Maqsood et al., 2022). Subcellular localization assays in tobacco leaves further support the idea that both *IpcHKT1;1* and *IpcHKT1;2* are membrane-bound transporters localized to the plasma membrane (**Figure 4**). However, the results also indicate that *IpcHKT1;2* contains additional short peptide chains at the N-terminus and longer peptide chains at the C-terminus compared to *IpcHKT1;1*, suggesting potential functional or regulatory differences between the two proteins (**Figure 2C**). These findings imply that the two HKT genes in *IPC* may have undergone functional differentiation. The divergence in their functions may be related to environmental selection pressures, as *IPC* (a species within the *Ipomoea* genus) grows in coastal environments and is exposed to extreme conditions such as high salinity and heat (Fita et al., 2015; Kotula et al., 2020). This likely led to the evolution of regulatory mechanisms to adapt to these challenging conditions. Additionally, this research provides a basis for improving the salt tolerance of *Ipomoea* species, such as sweet potato, through the incorporation of genes related to salt tolerance from *IPC*.

The expression of HKT genes is typically influenced by stress conditions, such as high sodium or low potassium concentrations (Huang et al., 2020; Huang et al, 2020; Jaime-Pérez et al., 2017; Su et al., 2003). However, there does not appear to be a universal pattern across species. For instance, high Na^+^ concentrations may upregulate the expression of certain HKT gene members while downregulating others (Jiang et al., 2018). Similarly, in some species, gene expression is upregulated in the shoots and downregulated in the roots, while the opposite effect is observed in other species (Oomen et al., 2012). This suggests that the expression and regulation of the HKT family is complex, providing plants with various mechanisms to adapt to stress conditions. The investigation of HKT expression responded to salt treatment in this study support also showed this tendency (**Figure 5**). Under normal conditions, the expression of *IpcHKT1;1* remained relatively stable across different tissues. However, under salt stress, its expression was downregulated in roots, stems, and leaves, followed by a gradual recovery. In contrast, under normal conditions, the expression of *IpcHKT1;2* was significantly higher in the stems (more than 20 times that in the roots) and in the leaves (more than 6 times that in the roots). After 4 hours of salt stress, the expression of *IpcHKT1;2* increased more than 40 times in the roots, with notable upregulation in both stems and leaves. After 72 hours, its expression remained high, suggesting that *IpcHKT1;2* may play a role in *IPC*’s long-term adaptation to high salinity. Additionally, when the expression of *IpcHKT1;2* began to decrease after 12 hours of salt stress, *IpcHKT1;1* expression began to recover across all tissues. This suggests that *IpcHKT1;1* and *IpcHKT1;2* may adopt distinct, possibly even antagonistic, strategies in response to salt stress, and there may be a regulatory interplay between these two genes in *IPC*.

To date, several plant *HKT* genes have been cloned and expressed in yeast systems to assess their selectivity in K^+^ and Na^+^ transport. Heterologous expression of *AtHKT1;1*, *HvHKT1;5*, *Ni-OsHKT1;5*, and *Po-OsHKT1;5* proteins in yeast has demonstrated their role as Na^+^/K^+^ symporters (Haro et al., 2005; Horie et al., 2001; Schachtman and Schroeder, 1994b). However, the heterologous expression of *OsHKT2;1* and *TaHKT2;1* results in Na^+^/K^+^ symport activity under low Na^+^ concentrations, but they function as Na^+^ transporters under high Na^+^ concentrations (Oomen et al., 2012). Additionally, *EcHKT1;1* and *EcHKT1;2* act as Na^+^/K^+^ symporters in yeast, even at elevated Na+ concentrations. Furthermore, Ca2^+^ and Mg2^+^ have been shown to also pass through EcHKT1;1 and EcHKT1;2 via membrane transport (Fairbairn et al., 2000; Liu et al., 2001). In *Ipomoea batatas* (sweet potato), the *IbHKT1* gene acts as a Na^+^/K^+^ symporter in yeast, and transgenic sweet potato roots overexpressing *IbHKT1* exhibit a significant increase in K^+^ uptake, suggesting that *IbHKT1* plays a role in K^+^ absorption in plants (Park et al., 2017). In the current study, heterologous expression of *IpcHKT1;1* and *IpcHKT1;2* in yeast defective strains (CY162 and AXT3K) revealed that these genes from IPC exhibit weak K^+^ transport activity and, in the presence of high Na^+^ concentrations, possess Na^+^/K^+^ symporter activity (**Figure 6**). These findings are consistent with those observed in sweet potato, suggesting that the functions of HKT proteins within the *Ipomoea* genus are relatively conserved.

## 5 Conclusion

In summary, we conducted a genome-wide identification of *HKT* genes in *IPC*, followed by a comparative analysis of *HKT* genes in representative species of the *Ipomoea* genus, including chromosomal localization, phylogenetic analysis, gene structure, protein structure, and cis-element analysis. The results revealed that *HKT* genes within the *Ipomoea* genus are highly conserved in terms of both their origin and function. Furthermore, we performed subcellular localization, salt stress response, and yeast functional complementation assays for the *IpcHKT* genes. These experiments demonstrated that *IpcHKT* genes are localized to the plasma membrane, function as Na^+^/K^+^ symporters, respond to salt stress, and play a crucial role in the salt tolerance of *IPC*. This study enhances our understanding of the evolutionary conservation of HKT gene structure and function in the *Ipomoea* genus, and provides new insights into the role of HKT genes in plant salt tolerance mechanisms.

## Data Availability

The genome assembly and annotation for Ipomoea pes-caprae were obtained from the National Genomics Data Center of China (https://www.cncb.ac.cn/) under BioProject ID PRJCA020559.

## Supplementary material

Supplementary Figure 1 Map of pENTER vector.

Supplementary Figure 2 Map of pCAMBIA2300-35S-EGFP-35S-Neo vector.

Supplementary Figure 3 Map of PYES2-NTB vector.

Supplementary Figure 4 Spatiotemporal expression profiling of *IpcSOS1;1* and *IpcSOS1;2* under salinity stress.

Supplementary Table 1 Sequences of primers used in this study.
