# Supplementary material for: Comparative analysis of HKT genes in Ipomoea pes-caprae unveils conserved Na+/K+ symporter functions within the gene family
Source: Front Plant Sci. 2025 Apr 1;16:1538669. doi: 10.3389/fpls.2025.1538669 (PMC12005088; doi:10.3389/fpls.2025.1538669)
Supplement: Supplementary Figure 1 — Map of pENTER vector. [file DataSheet1.pdf]

Supplementary materials

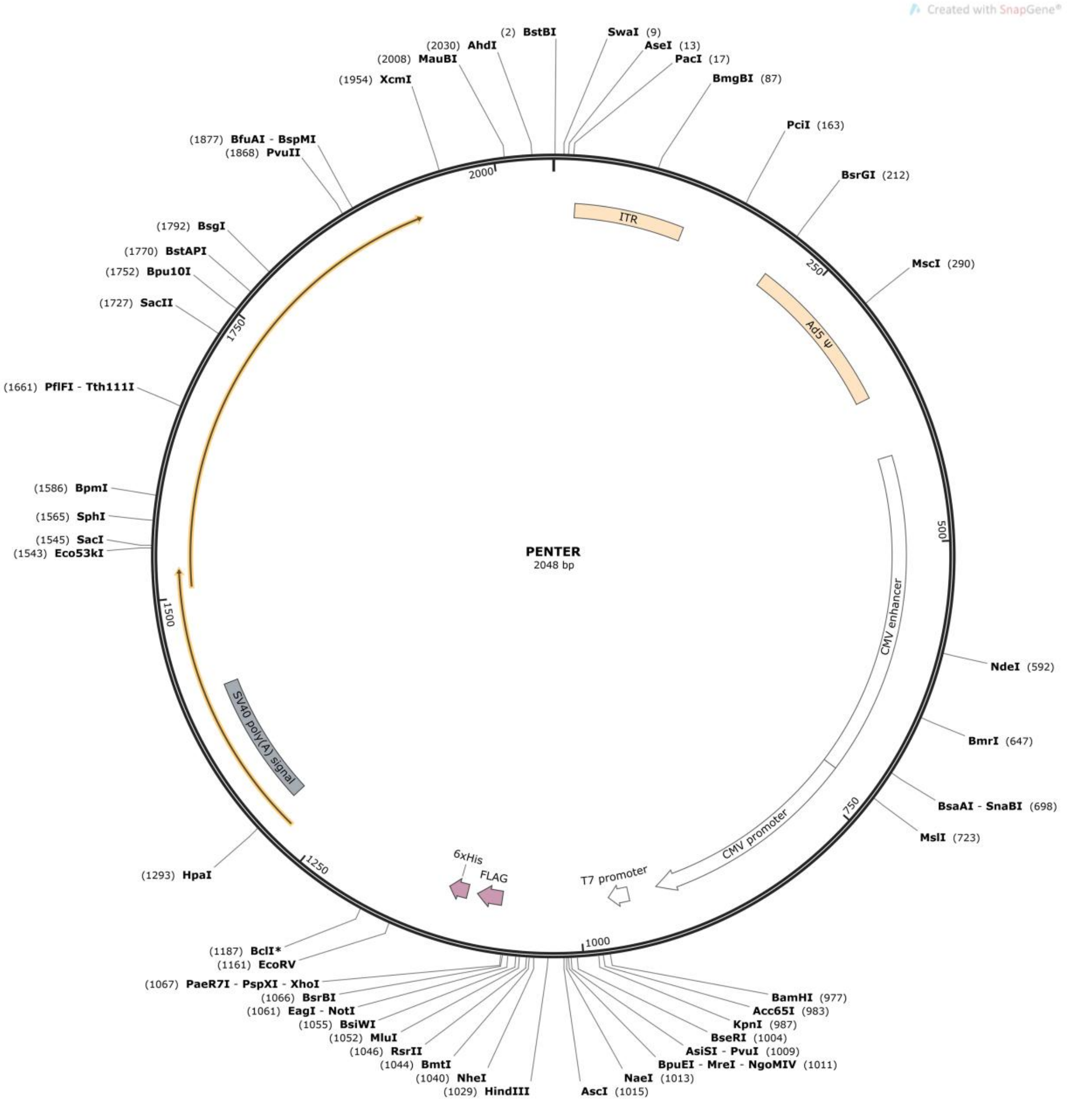

Figure S1. Map of pENTER vector.

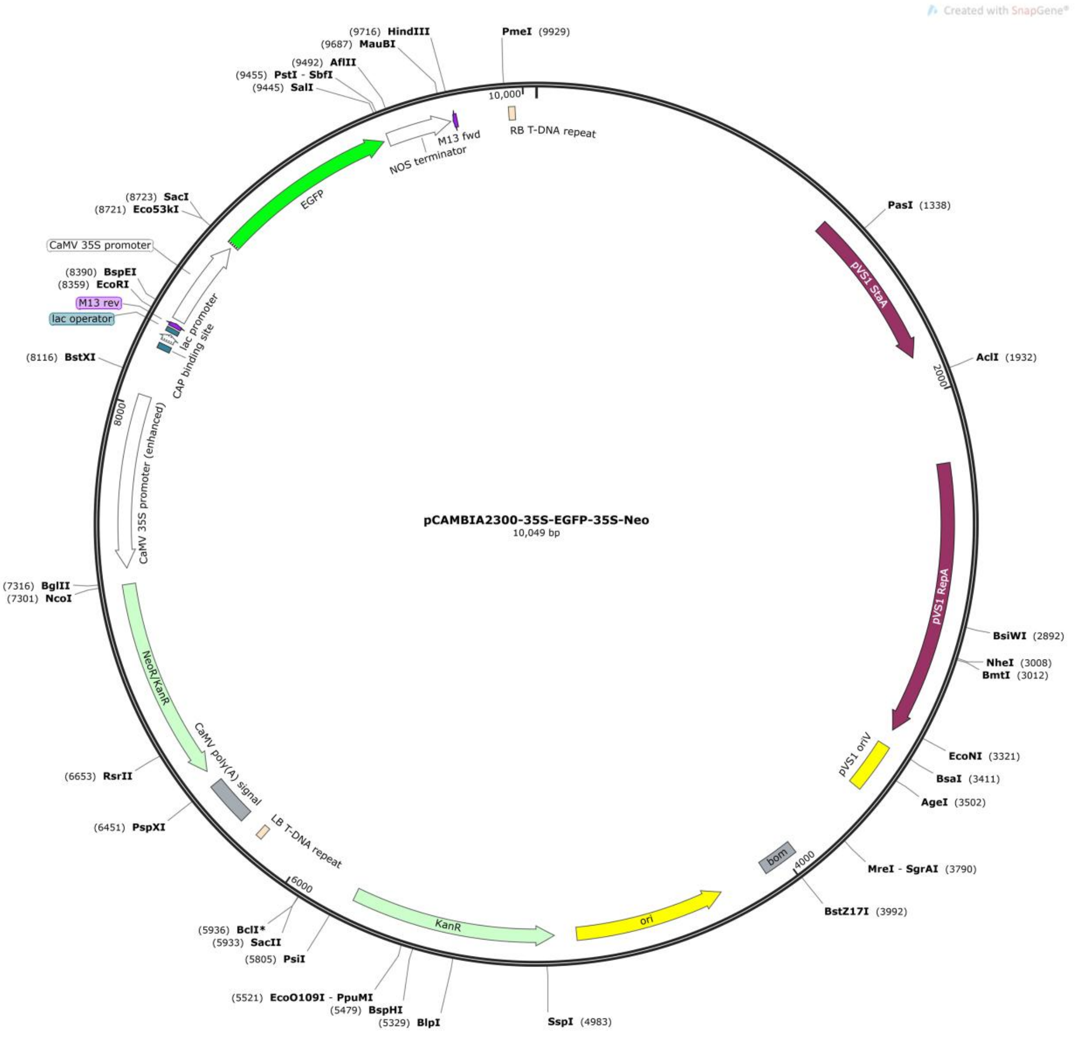

Figure S2. Map of pCAMBIA2300-35S-EGFP-35S-Neo vector.

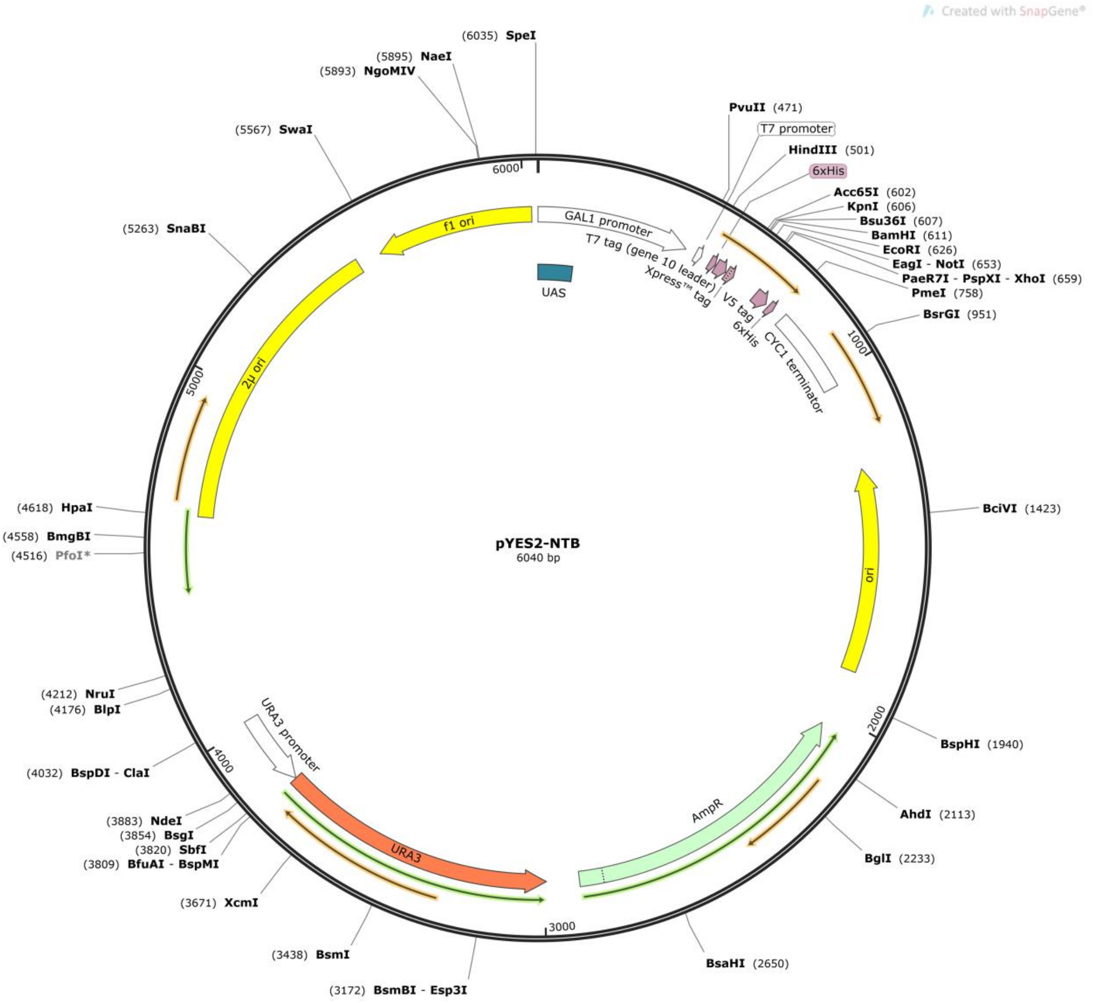

Figure S3. Map of PYES2-NTB vector.

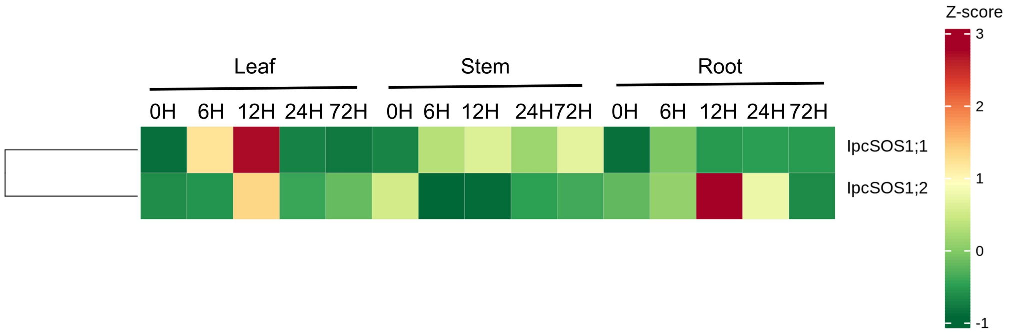

Figure S4. Spatiotemporal expression profiling of *IpcSOS1;1* and *IpcSOS1;2* under salinity stress.

Table S1. Sequences of primers used in this study.

Primer name	sequence
Q-IpcUBQF	TCGACAATGTGAAGGCAAAG
Q-IpcUBQR	CTTGATCTTCTTCGGCTTGG
Q-IpcHKT1；1-F	TCCTGGCTTGGGCTTCATTT
Q-IpcHKT1；1-R	GCCGTGGCGAGGTTTCTT
Q-IpcHKT1；2-F	ACTCCCCAAGTATAGGCCGT
Q-IpcHKT1；2-R	TTTGGGGACTCCTCGGGTAT
pCAMBIA2300-IpcHKT1；1-F	ATTTGGAGAGGACACGAATTCATGATGAGTGTCCGTAGGTTAGCG
pCAMBIA2300-IpcHKT1；1-R	AAGGGCTGCGGCCGCCTCGAGCCCAAACTGTTTATCTTTGCAATGGC
pCAMBIA2300-IpcHKT1；2-F	ATTTGGAGAGGACACGAATTCATGTTCCGGCCTAGGAATATCGAC
pCAMBIA2300-IpcHKT1；2-R	AAGGGCTGCGGCCGCCTCGAGTCAAATATAAGGACTAGGGTTCATTC
P-YES-LPCHKT1;1F	CGATGACGATAAGGTACCTAAGGATCCATGATGAGTGTCCGTAGGTTAG
P-YES-LPCHKT1;1R	CCACTGTGCTGGATATCTGCAGAATTCCCCAAACTGTTTATCTTTGCAATG
P-YES-LPCHKT1;2F	CGATGACGATAAGGTACCTAAGGATCCATGTCCACCACGGGGAGAAG
P-YES-LPCHKT1;2R	CCACTGTGCTGGATATCTGCAGAATTCTCATTGGATGACTGACTGTTC
P-YES-ATHKT1F	CGATGACGATAAGGTACCTAAGGATCCATGGACAGAGTGGTGGC
P-YES-ATHKT2R	CCACTGTGCTGGATATCTGCAGAATTCTTAGGAAGACGAGGGGTAAAG
